# The Pathogenic Features of Severe Acute Respiratory Syndrome Coronavirus 2 (SARS-CoV-2): Possible Mechanisms for Immune Evasion?

**DOI:** 10.3389/fimmu.2021.693579

**Published:** 2021-07-14

**Authors:** Zhihui Wang, Ming Zhou, Zhenfang Fu, Ling Zhao

**Affiliations:** ^1^ State Key Laboratory of Agricultural Microbiology, Huazhong Agricultural University, Wuhan, China; ^2^ Key Laboratory of Preventive Veterinary Medicine of Hubei Province, College of Veterinary Medicine, Huazhong Agricultural University, Wuhan, China

**Keywords:** SARS-CoV-2, pathogenic features, S protein, interaction, immune escape, epidemiology

## Abstract

Severe acute respiratory syndrome coronavirus type 2 (SARS-CoV-2) is a newly emerging, highly transmitted and pathogenic coronavirus that has caused global public health events and economic crises. As of March 4, 2021, more than 100 million people have been infected, more than 2 million deaths have been reported worldwide, and the numbers are continuing to rise. To date, a specific drug for this lethal virus has not been developed to date, and very little is currently known about the immune evasion mechanisms of SARS-CoV-2. The aim of this review was to summarize and sort dozens of published studies on PubMed to explore the pathogenic features of SARS-CoV-2, as well as the possible immune escape mechanisms of this virus.

## Introduction

Three types of coronaviruses have broken through the species barrier and led to lethal infectious pneumonia worldwide during this century. In November 2002, the first case known as severe acute respiratory syndrome (SARS) caused by SARS-CoV (1) occurred in Foshan, China; this virus has attracted much attention since it was first reported. Until July 2003, 8,096 cases, including 774 deaths, were reported in 27 countries. Since then, no additional infected cases have appeared (2), and the SARS-CoV disappeared from the public gaze. Considerable literature has grown around the theme of biodiversity and genomics of coronaviruses.

In June 2012, approximately 10 years after the first appearance of SARS-CoV, a new coronavirus known as Middle East respiratory syndrome coronavirus (MERS-CoV) was isolated from the sputum of a Saudi Arabian man who died of acute pneumonia (3). As of April 26, 2016, 1,728 cases, including 624 deaths, were reported in 27 countries. To date, MERS still exists in the Middle East and is one of the major public health problems there.

In December 2019, the first case of SARS-CoV-2 infection was reported in Wuhan, China, and viral genome sequencing and virus isolation were carried out against the clock by Chinese scientists in January 2020 (4). As of March 4, 2021, 114,653,749 people had been infected with the disease, resulting in 2,550,500 deaths in 223 countries and regions (5). SARS-CoV-2 has caused huge public health crisis as well as great social panic and worry. On 30 January 2020, the WHO Director-General Dr. Tedros Adhanom Ghebreyesus declared the COVID-19 outbreak a Public Health Emergency of International Concern.

In view of the death and economic loss worldwide caused by the SARS-CoV-2 pandemic, it is urgent to understand the viral biological characteristics and immune escape mechanisms. The main aim of this review is to summarize the interaction between SARS-CoV-2 and host cells by reviewing the published research on SARS-CoV-2 and systematically evaluate the possible mechanisms involved in host immune system escape to provide a theoretical basis for clinical treatment and disease control.

## Characteristics of SARS-CoV-2

Coronaviruses are a group of single-stranded RNA viruses that are widely distributed worldwide. The causative agent of COVID-19 was identified as a new type of coronavirus, probably having originated in animals (6, 7).

Coronaviruses have a single strand, positive sense RNA (SS+ RNA) genome that is the largest in RNA viruses, with a cap structure at the 5’ end and a polyA tail at the 3’ end (8). The genome of SARS-CoV-2 is arranged in the order of noncoding 5’-UTR-replicase (Orf1ab)-structural protein and nonstructural protein-noncoding 3’-UTR (9) (**Figure 2**). S, M, N and E open reading frames (ORFs) encode four kinds of structural proteins, while other ORFs encode nonstructural proteins. In addition, the SARS-CoV-2 has evolved an extremely CpG-defect genome to restrict zinc finger antiviral protein (ZAP) from combining with viral RNA (10).

The origin of SARS-CoV-2 remains a mystery. Regardless, finding patient zero is crucial to the source of SARS-CoV-2. Forster et al. analysed 160 nearly intact genomic datasets and divided them into types A, B and C. Based on this research, the phylogenetic network of SARS-CoV-2 was mapped (11). In their model, type A was divided into two subclusters by synonymous mutation T29095C. The data showed that the T allele subgroup in type A was distributed in China (n=4), Japan (n=3) and the United States (n=2), while the C allele subgroup had a long variation branch, and nearly half (15/33) of them were distributed mainly in the United States and Australia. Type B was mainly distributed in Asia (93/112), specifically in Wuhan (n=22), including other areas in eastern China (n=31) and 21 Asian countries adjacent to China. Type C was mainly prevalent in Europe (n=11) and was representative in France, Italy, Sweden, the United Kingdom, California and Brazil.

After more than a year of selective evolution, SARS-CoV-2 has developed a variety of different genotypes, including several types of superepidemic strains, as shown in full genome tree by GISAID (https://www.epicov.org/epi3/frontend#).

### Composition of the SARS-CoV-2 Particle

Coronavirus particles contains four kinds of structural proteins (12): a membrane glycoprotein, spike protein, nucleocapsid protein and envelope protein. The S-protein is the main fibril component and is a homotrimer. Each monomer contains a S1 subunit and a S2 subunit. The basic skeleton of coronavirus is constituted by the M-protein and E-protein, and RNA is embedded in the N-protein to form the nucleocapsid. The S-trimers on the surface of the virus present in two different forms: a few of them are long and thin, similar to the prefusion form, while others are wider, similar to the postfusion form. S-trimers with different conformations are evenly distributed on the virus surface (13).

Coronavirus enters host cells through the S protein on its surface (14). Therefore, the S-protein goes hand in hand with the virulence and invasion of coronavirus. In most cases, the S-protein is cut into S1 and S2 subunits by host proteases, which are responsible for receptor recognition and membrane fusion (15). The S1 subunit contains an N-terminal domain (NTD) and a C-terminal domain (CTD), both of which can bind to receptors. For example, SARS-CoV and MERS-CoV use the S1 CTD to identify receptors (16, 17).

After the outbreak of COVID-19, scientists quickly determined that SARS-CoV-2 entered host cells *via* the hACE2 receptor (4). Considering SARS-CoV using S1 CTD to recognize the hACE2 receptor, Wang et al. revealed the structural basis of SARS-CoV-2 recognition and binding to receptors using immunofluorescence staining and flow cytometry (18). They found that the S1 CTD was the key region that interacts with the hACE2 receptor in SARS-CoV-2.

#### Structural Basis of SARS-CoV-2 S Protein

**Figure 1:**
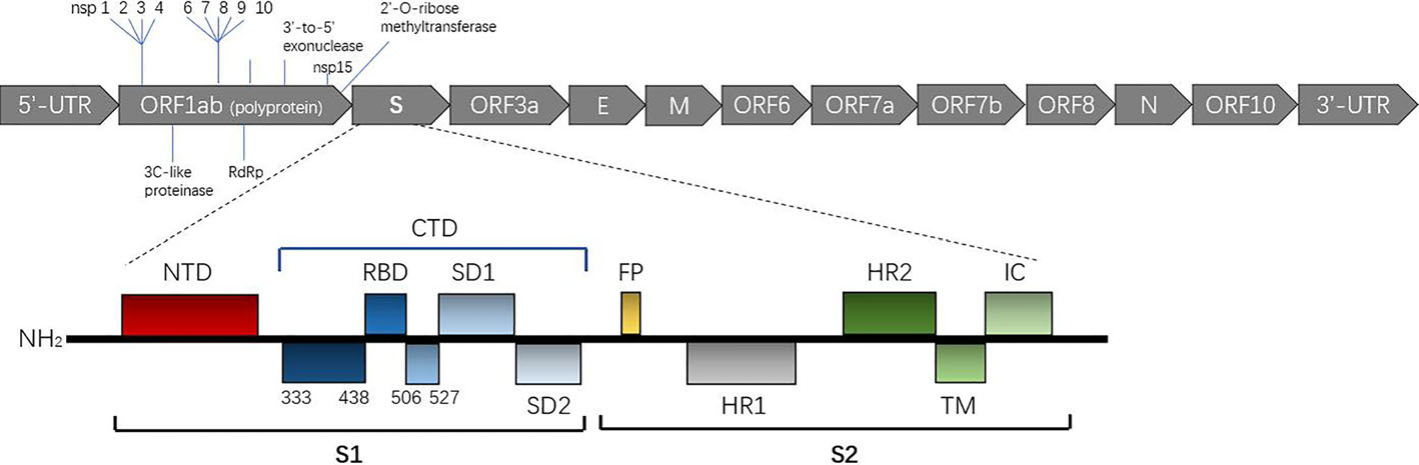
Overall topology of the SARS-CoV-2 spike monomer. The secondary structure of the spike monomer contains two parts, S1 and S2. S1 is composed of an NTD (shown in black) and CTD (shown in blue) containing the receptor binding domain (RBD) necessary for hACE2 binding. (Expasy Reference Sequence: P0DTC2).

Bioinformatics analysis showed that SARS-CoV-2 S-protein had typical characteristics of that for coronavirus and is composed of an S1 subunit containing an NTD and CTD, S2 subunit, transmembrane domain and short intramembrane domain, as shown in **Figure 1**. Phylogenetic analysis showed that the S-protein of SARS-CoV-2 shares ~ 77% homology with that of SARS-CoV and 96.2% homology with that of a bat coronavirus named BatCoVRaTG13 (4). This finding suggests that the ancestors of SARS-CoV-2 may have been derived from bats. Interestingly, a furin-like protease site considered to have the ability to promote viral fusion with cell membranes is reportedly on the boundary of the S1/S2 subunit (19, 20).

**Figure 2:**
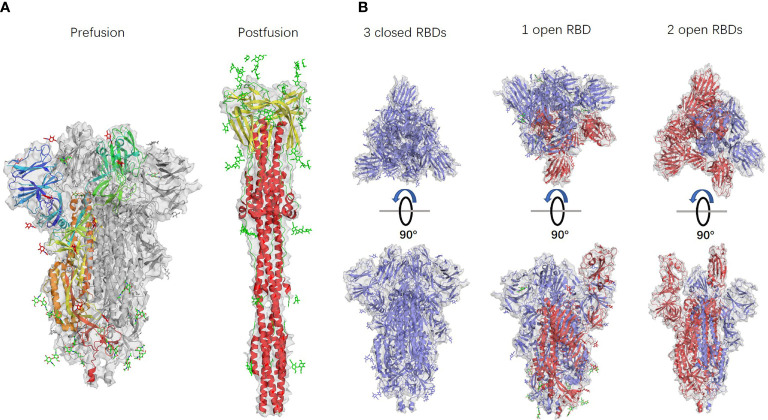
Different S-trimer conformations. PDB accession numbers: 6 VXX, 6 XRA, 6 VXX, 6 VYB, and 6 X2B from left to right. **(A)** S-trimer models in prefusion and postfusion forms. **(B)** S-trimer models with different RBD forms. S-monomers with closed RBDs are shown in blue, and open RBDs are shown in red.

Ke et al. identified 4220 S-trimers from 179 virus particles isolated from the supernatant of infected cells and analysed them by subtomogram averaging to identify the S-trimers structure. The authors found different types of conformations: among all 4200 S-trimers, 4104 (approximately 97%) were in the prefusion conformation, and 116 (approximately 3%) were in the postfusion conformation. Among all 3854 prefusion S-trimers, the proportion of trimers with all three closed RBDs was 31%, and the proportion of trimers with one open RBD was 55%, while the remaining 14% had two open RBDs (13). All the conformations are shown in **Figure 2**. This research suggests that in the absence of ACE2, there may be both closed and open S-trimers on the surface of SARS-CoV2 particles. The prefusion trimers may be dominated by closed conformations, while the open conformation is induced or stabilized only when bound to ACE2.

## Interaction Between SARS-CoV-2 and the Human Body

Pathogenic factors can cause damage to the body. Several defence and compensatory functions in the body are mobilized to fight against pathogenic factors and the resulting damage. SARS-CoV-2 has a series of mechanisms to escape the host immune system; however, similar to SARS, some patients with COVID-19 experience inflammatory storms caused by excessive cytokines in the late stage (21), especially a series of chemokines in the plasma, such as CXCL10, IP-10, CCL2, MCP-1, MIP-1a/CCL3 and TNF-α, which increase uncontrollably (22) and lead to acute respiratory distress syndrome (ARDS) and poor prognosis. Therefore, whether immunosuppression is needed to address a patient’s excessive inflammatory response in clinical treatment is unclear (23).

The above findings may suggest that the immune-escape effect of SARS-CoV-2 is mainly reflected in the early stage of the disease, which leads to the virus replicating further in the host body. At present, data about the immune evasion mechanisms of SARS-CoV-2 are limited. This chapter seeks to explain the development of SARS-CoV-2 based on speculation and published literature to provide a theoretical basis for clinical treatment and related scientific research.

### High Affinity of S Protein With hACE2

The Lan laboratory is one of the first research teams to determine the crystal structure of the S protein RBD. The structure of the SARS-CoV-2 RBD-ACE2 complex was successfully determined by X-ray crystallography, and the residues in the SARS-CoV-2 RBD that are essential for binding ACE2 were identified (24). Most of the RBD residues in the S protein were highly conserved and similar in side chain characteristics compared with those of the SARS-CoV RBD. The authors predicted that these two viral antigens might have similar affinity to hACE2.

**Figure 3:**
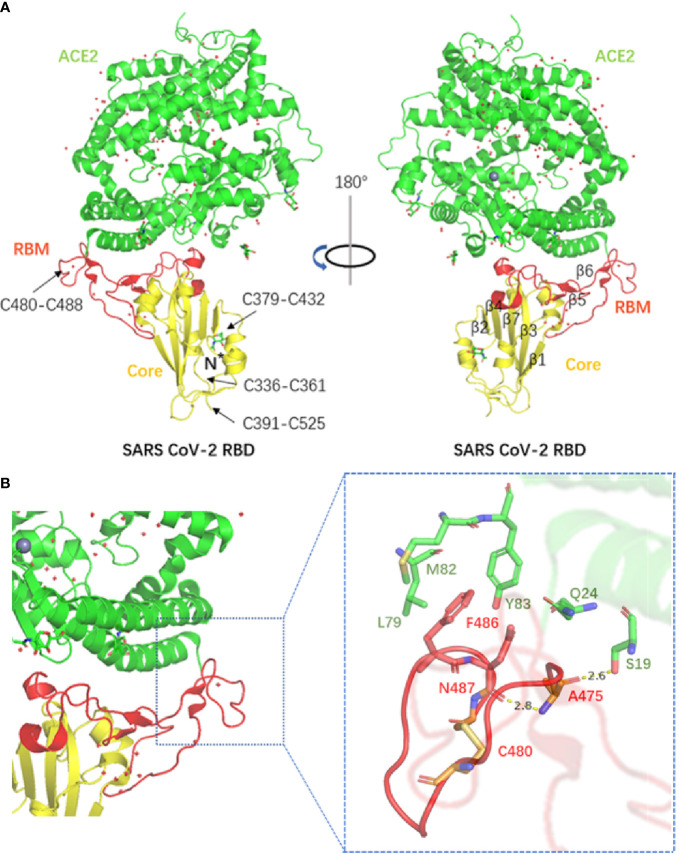
**(A)** The structure of the SARS-CoV-2 RBD complex bound to ACE2. Disulphide bonds in the SARS-CoV-2 RBD are indicated by arrows (PDB accession number: 6 M0J). **(B)** The structure of the SARS-CoV-2 chimeric RBD complexed with hACE2. The hydrogen bond as well as parts of the interactions are shown in the figure (PDB accession number: 6 VW1).

Although the homology of the S protein between SARS-CoV-2 and SARS-CoV seems to be high, a series of subsequent reports have challenged the significance. Researchers found that the SARS-CoV-2 S protein had a higher affinity for the hACE2 receptor than SARS-CoV. Compared with the SARS-CoV receptor binding motif (RBM), SARS-CoV-2 forms a larger binding interface and more connections with hACE2. In addition, a significant difference in the structure of the RBM between SARS-CoV-2 and SARS-CoV was recognized, as the backbone of the SARS-CoV-2 RBM forms more contacts with the N-terminal helix of hACE2 than that of SARS-CoV, resulting from the difference in the ring conformation on the binding backbone. Furthermore, compared with the leu472 site of the SARS-CoV RBM, the phe486 site of the SARS-CoV-2 RBM points in different directions and possibly has the ability to insert into the hydrophobic pocket of met82, leu79 and tyr83 of hACE2 (25). The structures of the SARS-CoV-2 RBD complex bound to ACE2 and the SARS-CoV-2 chimeric RBD complexed with hACE2 are shown in **Figure 3**. Therefore, we believe that the SARS-CoV-2 S protein has more atomic interactions.

The data from Wang et al. also support this point of view. The equilibrium dissociation constants (K_D_) of SARS-CoV-2 S1 and CTD binding hACE2 were 94.6 ± 6.5 nm and 133.3 ± 5.6 nm, respectively, as determined by real-time surface plasmon resonance (SPR) analysis (18), which indicated that the affinity of SARS-CoV-2 with the same receptor was approximately 4 times higher than that of SARS-CoV, of which the K_D_ was 408.7 ± 11.1 nm.

As a result of the interspecific specificity of ACE2, the binding affinities of SARS-CoV-2 to the ACE2 receptor from different species remain different. For example, SARS-CoV-2 has a relatively higher affinity for the ACE2 receptor of humans, nonhuman primates and cats than other species (26). In most cases, the binding affinity between the S protein and the hACE2 receptor is higher than that of other interacting proteins in nature (27), including one of the antibodies with the highest affinity (anti-TSH-R, 7.2E-09 mkd), which is much stronger than that of the T cell receptor (TCR) with a major histocompatibility complex (MHC) (2.00e-06 mkd).

Therefore, to what extent the high affinity between the SARS-CoV-2 S protein and hACE2 may interfere with the neutralization effect of anti-S immunoglobulin and lead to immune escape is unclear. As mentioned above, the RBDs of the S protein are mostly in the “down” conformation (not open) when not bound to ACE2. We speculate that this property may interfere with the production, recognition and binding of specific antibodies to a certain extent.

### RNA Genome Modification by Nonstructural Proteins

The 5’ cap structure is a unique feature of eukaryotic mRNA. Some types of viruses are able to modify the 5’ end of their nucleic acids to mimic the structure of cellular mRNA, which is crucial for RNA stability, protein translation and immune escape. Previous studies have shown that 2’-*O*-methylation of the viral RNA cap sequence can help viruses escape host immune recognition (28). Therefore, we focused on the possible genome modification mechanism of SARS-CoV-2.

Chen et al. successfully studied the structure of the nuclease P1-resistant cap released by P_32_-labeled G*pppA RNA and performed TLC analysis of m7G*pppA RNA methylated by different types of nsp protein combinations. In addition, the authors also detected the methylation activity of the nsp16/nsp10 complex from SARS-CoV with different RNA substrates and that of nsp16/nsp10, nsp16 and nsp10 with P_32_-labeled G*pppG RNA as substrates (29, 30). Through gene screening and biochemical analysis, two nonstructural proteins encoded by SARS-CoV with S-adenosylmethionine (SAM)-dependent methyltransferase (MTase) activity, nsp14 and nsp16, were found.

In contrast to nsp14, the catalytic activity of nsp16 also depends on the stimulating subunit nsp10. Nsp14 N7-MTase can make RNA with the cap sequence of GppppA- and GpppgG- methylation without sequence specificity, while the nsp16/nsp10 complex only actuates the strong signal of methylation on m7GpppA modified RNA substrate, which indicates that the nsp16/nsp10 complex acts depending on the specificity of the cap sequence.

Based on previous reports, the compositing path of SARS-CoV-2 RNA CAP synthesis is shown. First, the terminal γ-phosphates are removed from the 5’-triphosphate end of the RNA by RNA 5’-triphosphatase (RTPase). Second, GMP molecules are added to the 5’-RNA to form the GpppN structure by RNA guanosine transferase (GTase). Third, GpppN-RNA is methylated at the N7 position of guanosine by N7-MTase (nsp14) to produce m7GpppN-RNA, known as cap-0. Finally, the 2’-*O* site of the first ribonucleotide in m7GpppN is methylated to synthetize m7GpppNm-RNA by the nsp16/nsp10 complex (cap-1).

In conclusion, the nsp14 and nsp16/nsp10 complex can avoid recognition of exogenous RNA by the host innate immune response by catalysing 2’-*O*-methylation of the cap sequence of the SARS-CoV RNA genome. Based on the findings above, the same escape mechanism may exist in the process of SARS-CoV-2 infection (31).

### Interference With the Host Biological Activities and Inhibition of Signalling Pathways

The innate immune system recognizes pathogen-associated molecular patterns (PAMPs) by pattern recognition receptors (PRRs) and then initiates a series of downstream signalling pathways, resulting in cascade-effects releases of various cytokines, including interferon (IFN) family. The induction of IFN is one of the main innate antiviral defence pathways in the host body. Several proteins encoded by coronavirus members have multiple strategies to antagonize innate immunity, especially IFN signalling pathway. The pandemic of COVID-19 gives urgency to constitute an integrated understanding of the interference of SARS-CoV-2 with host signalling pathways. Furthermore, referring to previous studies on SARS-like CoVs, SARS-CoV-2 may have an immune escape mechanism similar to that of other coronaviruses. Thus, reviewing that of SARS and MERS is still necessary for the understanding of COVID-19 as a reference. Here, we summarize the interference with host biological activities and inhibition of signalling pathways in course of COVID-19.

#### Interfere With IFNs Upstream Pathway

After binding to hACE2, the virus enters the host cell and replicates in its cytoplasm. The RIG-1-likereceptor (RLRs) including retinoicacidinduciblegene-1 (RIG-1) and melanoma differentiation-associated gene-5 (MDA5) in the cytoplasm can recognize viral dsRNA and Toll-like receptors (TLRs) including TLR-3, TLR-7 and TLR-8 on the membrane of endosome can recognize viral ssRNA and dsRNA, leading to downstream signalling pathway and resulting in IFNs generation.

**Figure 4:**
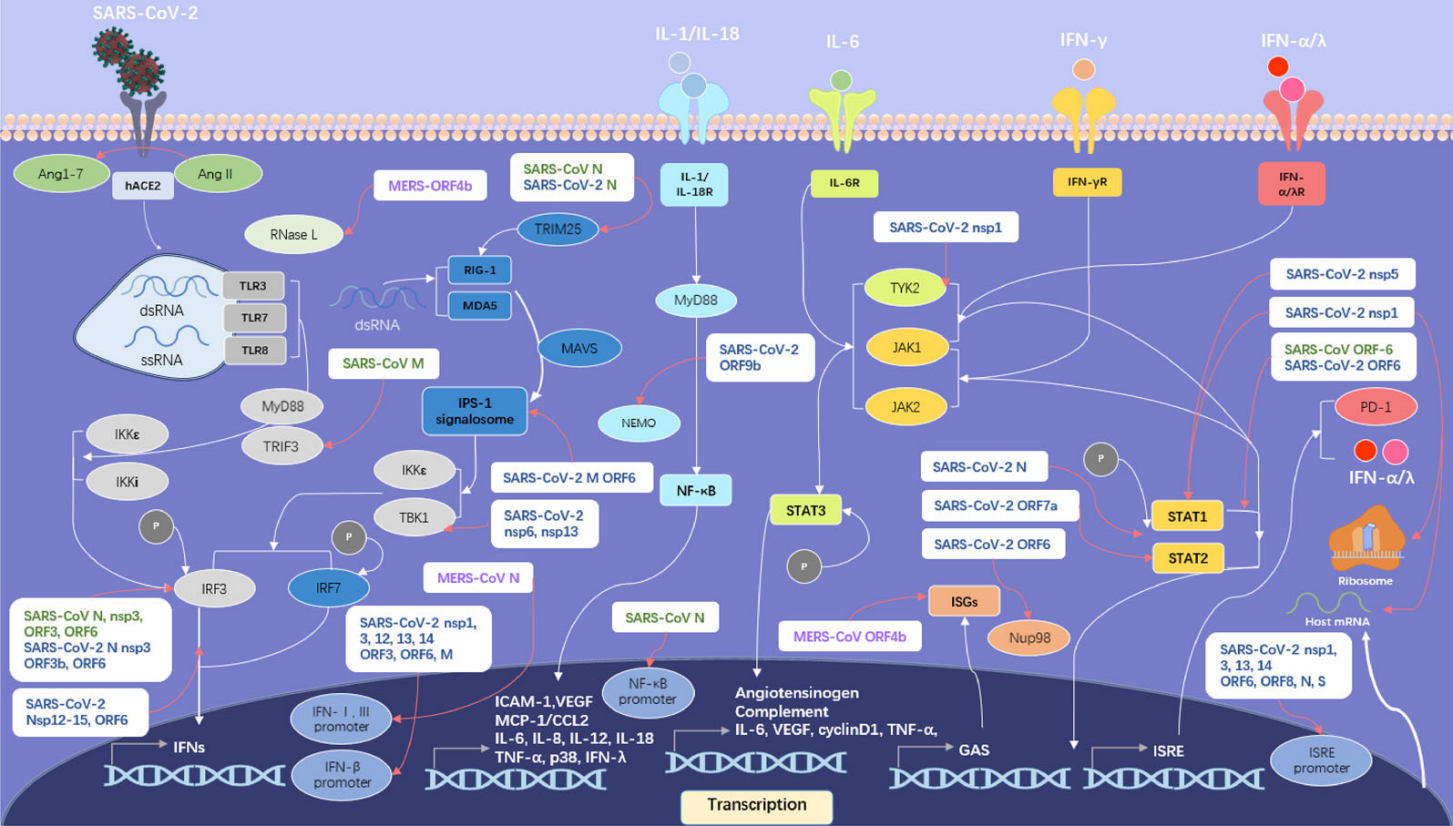
SARS-like CoVs including SARS-CoV, MERS-CoV and SARS-CoV-2 inhibit host IFN signalling pathway. Proteins of SARS-CoV are in green, proteins of MERS-CoV in violet and proteins of SARS-CoV-2 in blue. The IFN upstream pathways are in the left side of the figure and the IFN downstream pathways are in the right side. The pattern diagram of SARS-CoV-2 is credited by Maria Voigt/RCSB PDB.

Previous studies have revealed that SARS-CoV N, nsp3, ORF3 and ORF6 proteins can inhibit the phosphorylation of interferon regulatory factor 3 (IRF3) (32, 33) and the SARS-CoV-2 has the similar ability to inhibit IFNs upstream pathway using several strategies including that shown in **Figure 4**.

SARS-CoV-2 ORF3b protein antagonizes IFNs potently (34), possibly by inhibiting the phosphorylation of IRF3. SARS-CoV-2 ORF6 protein interferes with the assembly of interferon-β promoter stimulator-1 (IPS-1) signalosome and blocks IRF3 activation (35). The ORF6 protein also binds to importin Karyopherin α 2 (KPNA2) and Nup98-Rae1, which is essential for IRF3 nuclear translocation, to interfere with IFN upstream pathway (36, 37). The ORF9b protein interrupts the K63-linked polyubiquitination of NEMO, an essential modulator in nuclear factor kappa-B (NF-κB) signalling pathway, to inhibit IFN production (38).

The papain-like protease (PLpro) in nsp3 protein of SARS-CoV-2 cleaves interferon-stimulated gene 15 (ISG15) from IRF3, prompting the attenuation of type I IFN responses (39), while Yuen et al. argued that PLpro didn’t have that function of IFN antagonists (40). The nsp5 protein can interact with RIG-I to reduce its K63-linked ubiquitination and restrain the Tank-binding kinase 1 (TBK1) and IRF3 phosphorylation. The nsp6 protein targets TBK1 to suppress IRF3 phosphorylation (36). Protein interaction map suggests that the SARS-CoV-2 nsp13 protein may have the ability to interact with TBK1 and reduce its activity (41), consistent with Xia’s result (36). Yuen et al. suggested the function of nsp13, nsp14, nsp15 to quell IRF3 nuclear localization (40) and further studies are required to elucidate those mechanisms.

The N protein in SARS-CoV-2 inhibits the interaction between tripartate motif protein 25 (TRIM25) and RIG-I (42), possibly using a strategy similar with SARS-CoV. Furthermore, the N protein is suggested to have the ability to interfere with RIG-I (43). In addition, the N protein has the ability to restrain the combination between TBK1 and IRF3, resulting in the failure of IRF3 nuclear translocation (42), while nsp12 protein just inhibits the nuclear translocation of IRF3 without interfering IRF3 phosphorylation (44).

The M protein in SARS-CoV-2 has an inhibitory effect on IFN-β production through blocking RIG-I and MAVS related to its mitophagy induction (45). In addition, the M protein prevents the formation of IPS-1 signalosome *via* interacting with RIG-I, MAVS, and TBK1 (46). Recent research suggests that the nsp1 protein variant with deletion in site Δ500-532 induced a decreased production of IFN-β in patient serum (47).

#### Interfere With IFN Downstream Pathway

Through autocrine and paracrine, type I and III IFNs induce a large number of antiviral effectors. However, SARS-like CoVs have evolved multiple strategies to inhibit IFNs downstream pathway as shown in **Figure 4**.

SARS-CoV-2 nsp1 protein can interact with Tyrosine Kinase 2 (TYK2) to inhibit IFN-α/λ downstream pathway (48). The nsp5 protein can interact with signal transducer and activator of transcription 1 (STAT1) and decrease the levels of this activator, possibly by contributing to its autophagic degradation (49).

SARS-CoV-2 N protein is able to interfere with the phosphorylation of STAT1 and STAT2 to suppress IFN-α/λ/γ downstream pathway (50). Significantly, the ability to interfere with the phosphorylation of STAT1 of SARS-CoV-2 in monocyte-derived dendritic cell (moDCs) is much stronger than that of SARS-CoV (51).

SARS-CoV-2 as well as SARS-CoV ORF6 protein can block the nuclear translocation of STAT1 (37), which may result in the disordered expression of ISGs (52). The ORF7a protein polyubiquitinated at Lys119 *via* the ubiquitin system in host cells gets the ability to inhibit type-I IFN downstream signalling pathway through restrain the STAT2 phosphorylation (53).

#### Interfere With Host Biological Activities

Besides inhibiting IFNs signalling pathway, SARS-like CoVs also have the ability to interfere with the translation, transcription and autophagy process of host cells. Previous research has established that N protein in SARS-CoV inhibited expression of an NF-κB-responsive promoter remarkably (33) and nsp-1 protein in SARS-CoV boosting host mRNA degradation as well as inhibiting translation resulting in restraining gene expression in host cell (54, 55). As shown in **Figure 4**, multiple proteins in SARS-CoV-2 inhibit the activation of IFN-β and ISRE promoter activity (35). Noteworthily, the recent study demonstrated that the nsp1 protein of SARS-CoV-2 could bind to 40S and 80S ribosomes and interfered with cap-dependent translation in host cells (56). And the key residue in nsp1 to inhibit host gene expression, a lysine, has been proven to be located at amino acid site 164 (K164) (57).

Moreover, SARS-CoV-2 has the ability to interfere with autophagy in host cells. While the ORF3a and ORF7a proteins counteract autophagosome activity, the nsp 14 protein interacts with type I IFN receptor, resulting in its lysosomal degradation (58). Noteworthily, the ORF8 protein interacting with MHC-I molecules is necessary for cellular immunity and may lead to the autophagy and down-regulating of MHC-I molecules through lysosomal degradation in ORF8-expressing cells, resulting low sensitivity for SARS-CoV-2 to cellular immunity by interfering cytotoxic T lymphocytes in COVID-19 patients (59).

### Invasion of the Blood-Cerebrospinal Fluid Barrier

The central nervous system (CNS), including the brain, is an immune-privileged site that is usually a reservoir for several viruses. Neurotropic viruses, including rabies virus (RABV) and Japanese encephalitis virus (JEV), are able to invade into and infect the CNS to induce neurological symptoms. As reported by Helms et al., neurological symptoms were observed in all 64 patients with acute respiratory distress syndrome (ARDS) associated with encephalopathy, obvious agitation, psychosis, and corticospinal tract symptoms due to COVID-19 (60). SARS-CoV-2 was detected in brain tissue samples from 22 patients who died for COVID-19 (61). Increasing clinical data suggest that SARS-CoV-2 may possess the ability to infect the CNS. To verify this hypothesis, Jiang et al. constructed a batch of HFH4-hACE2 transgenic mice and artificially infected them with SARS-CoV-2. The results demonstrated that SARS-CoV-2 viral RNA was detected in the brains of four out of twenty mice, and all these four mice died for COVID-19 (62).

Pellegrini et al. found that among different parts of the human brain, the level of ACE2 in the choroid plexus (CHP) was the highest, especially in CHP cells with a high level of lipoprotein expression (63). Further study showed that SARS-CoV-2 could infect CHP cells, but the susceptibility of nerve cells to SARS-CoV-2 was relatively low. In a brain-organ-like model developed by researchers, SARS-CoV-2 could destroy the integrity and barrier function of the CHP epithelium. Because the CHP is an important component of the blood cerebrospinal fluid barrier (B-CSF-B), the neurological symptoms caused by COVID-19 may be the result of the virus infection of Sertoli cells rather than neurons in the brain. I Above studies suggest that SARS-CoV-2 can invade into the brain to escape the host immune response.

### Antigenic Shift

Compared with DNA viruses, RNA viruses lack polymerase with error correction in the genome, which leads to more gene mutations and antigenic shifts. In addition, inadequate immune response or treatment with a high dosage of convalescent plasma as well as antiviral drugs may result in evolutionary processes or selective pressures on viruses in the host body. As mentioned above, SARS-CoV-2 has developed a variety of different genotypes after more than a year of selective evolution, including B.1.1.7 and B.1.351, which are two of the most concerning strains due to their increased infectivity.

Lineage B.1.1.7 was first detected in Britain in December 2020 (64). This variant contains seventeen nonsynonymous mutations, especially significant in its S protein, including mutation N501Y, spike deletion 69-70del and mutation P681H, which causes enhanced ACE2 receptor affinity and promotes entry into respiratory epithelial cells (65, 66). Lineage B.1.1.7 has rapidly risen to dominance in Britain (67).

Lineage B.1.351 was detected after the first severe COVID-19 pandemic in Nelson Mandela Bay located on the coast of the Eastern Cape Province, South Africa. This variant contains nine lineage-defining mutations in the S protein, including K417N, E484K and N501Y in its RBD (68), which may have biological significance (69); however, in view of the fact that the current vaccines mainly utilize ancestral SARS-CoV-2 S protein, the extent to which these mutations can affect SARS-CoV-2 immune escape remains to be investigated.

A growing body of research has demonstrated the immune escape effect of these two variants. Shen et al. found that B.1.1.7 remained sensitive to neutralizing antibodies in serum samples from convalescent individuals as well as recipients of a mRNA vaccine (mRNA-1273, Moderna) and a protein nanoparticle vaccine (NVX-CoV2373, Novavax) (67), while Collier et al. reported that mRNA-based vaccine (BNT162b22) serum was modestly reduced against the B.1.1.7 variant in neutralizing titres compared with wild-type pseudoviruses (70). Nevertheless, B.1.351 appears to show a much stronger escape effect. Wibmer et al. found six representative antibodies from class I with epitopes centred on spike residue K417, class II revealed key interactions with spike residue E484 that failed to bind 501Y. V2 RBD, and nearly half (21 of 44, 48%) of the serum samples from convalescent individuals had no detectable neutralization activity to B.1.351 (71). The findings of Wang et al. are in agreement, confirming that B.1.351 is a great threat to mAb therapy and the protective effect of the present vaccines (72).

Nucleotide mutations in the genome of circulating SARS-CoV-2 lineages have been reported to accumulate at a speed of approximately 1-2 mutations per month (73). In addition, 5 (codons 18, 80, 215, 484, 501) of 8 polymorphic spike gene sites from 142,037 high-quality sequences from 24 December 2019 to 14 November 2020 showed positivity of diversifying selection (68). Therefore, the evolution of the virus antigen is urgent to understand and corresponding prevention and control strategies must be formulated to address the emerging mutants.

## Perspective

The immune escape effects of pathogenic viruses have always been a hot issue for epidemiologists. Especially in the case of new outbreaks, aetiology and immunology studies play a crucial role in an epidemic response. In reviewing previous studies, few data have attributed the affinity between viral ligands and cell receptors as one of the immune indices; however, in view of the strong interaction between the SARS-CoV-2 S protein and hACE2, whether the interaction effect will interfere with the host’s immune response is unclear. Therefore, the use of multiple types of monoclonal antibodies may work better in clinical treatment.

In addition, as mentioned above, vaccination provides a useful back-up to prevent and control COVID-19 considering the strong ability to inhibit host innate immunity; however, large-scale application of vaccines may also aggravate the selective pressure on SARS-CoV-2, leading to the emergence of new genotypes and failure of existing vaccines. Therefore, further bioinformatics analyses and epidemiological investigations will need to take these variables into account.

SARS-CoV-2 evades the innate immune system *via* various strategies to antagonize IFN pathways and interfere the host’s normal physical activity. SARS-CoV-2 has a strong inhibitory effect on type-I IFN production while SARS-CoV-2 is much more sensitive to IFN-I than SARS-CoV (74). It is worth noting that the SARS-CoV-2 has the ability to infect moDCs and macrophages (MDMs), while none virus replication but viral protein production could be observed, suggesting a recessive infection in moDCs and MDMs and this possibly leads to recurrence of COVID-19 (51). Notably, this effect may be aggravated by antibody-dependent cell-mediated phagocytosis (ADCP) observed recently in several types of variants with D614G site (75).

The strong inhibitory effects against the host immune system may lead to defective clearance of the SARS-CoV-2, resulting in a persistent hyperactivation of the monocyte and macrophage as a compensatory mechanism. In the late stage of COVID-19, the symptoms of the disease manifests including Macrophage Activation Syndrome (MAS) and secondary Hemophagocytic Lymphohistiocytosis (HLH) as well as a poor number of several types of lymphocytes including CD4^+^, CD19^+^ and NK lymphocytes with cytokine storm containing IL-1, IL-6 and IL-10 (48, 76). The inflammatory over-reaction, as well as the disorder of renin-angiotensin system (RAS) resulted from the interaction between SARS-CoV-2 S protein with hACE2 (77), may give adverse effects on host immune system and contribute to virus proliferation. However, further studies are need to understand the causal link between the mechanisms for immune evasion with inflammatory storm caused by SARS-CoV-2.

To date, the SARS-CoV-2 epidemic continues worldwide and is related not only to the high transmission of the virus but also to the epidemic prevention measures of governments and citizenship. Compared with SARS-CoV, similarities exist in many physiological characteristics, such as receptor selection and clinical symptoms; however, a series of mutations of SARS-CoV-2 have greatly enhanced its propagation ability. In the history of confrontation between humans and pathogens, immune evasion and host immunity have formed a dynamic balance. In this review, we summarized the interaction between SARS-CoV-2 and the host as well as possible immune escape mechanisms of SARS-CoV-2, which provides a theoretical basis for clinical treatment and the design of novel drugs.

## Author Contributions

Drafting the manuscript: ZW. Revising the manuscript critically for important intellectual content: LZ, MZ, and ZF. All authors contributed to the article and approved the submitted version.

## Funding

This work was partially supported by the National Natural Science Foundation of China (Grant numbers 31872451 to LZ; 31720103917 to ZF).

## Conflict of Interest

The authors declare that the research was conducted in the absence of any commercial or financial relationships that could be construed as a potential conflict of interest.
